# Biofilms: hot spots of horizontal gene transfer (HGT) in aquatic environments, with a focus on a new HGT mechanism

**DOI:** 10.1093/femsec/fiaa031

**Published:** 2020-02-28

**Authors:** Kimihiro Abe, Nobuhiko Nomura, Satoru Suzuki

**Affiliations:** 1 Faculty of Life and Environmental Sciences, University of Tsukuba, Tsukuba, 305-8577 Japan; 2 Microbiology Research Center for Sustainability, University of Tsukuba, Tsukuba, 305-8577 Japan; 3 Center for Marine Environmental Studies, Ehime University, Matsuyama, 790-8577 Japan

**Keywords:** antibiotic resistance gene, horizontal gene transfer, membrane vesicle, biofilm, water environment

## Abstract

Biofilms in water environments are thought to be hot spots for horizontal gene transfer (HGT) of antibiotic resistance genes (ARGs). ARGs can be spread via HGT, though mechanisms are known and have been shown to depend on the environment, bacterial communities and mobile genetic elements. Classically, HGT mechanisms include conjugation, transformation and transduction; more recently, membrane vesicles (MVs) have been reported as DNA reservoirs implicated in interspecies HGT. Here, we review the current knowledge on the HGT mechanisms with a focus on the role of MVs and the methodological innovations in the HGT research.

## Teaser

Biofilms and membrane vesicles are the stage of gene transfer.

## INTRODUCTION

Based on surveillance data, the acquisition of antibiotic resistance genes (ARGs) by pathogens in natural environments is expected to become increasingly severe, expanding across multiple countries with variations in the population of bacteria that are resistant to drug treatment (Hashiguchi *et al*. 2019). Like clinical settings, the natural environment should be a focus of attention targeting the control of antibiotic-resistant bacteria (ARB) and ARGs.

When we look at the natural environment, ARB and ARGs can be detected in environments without selective pressure (Sizemore and Colwell 1977), including the open ocean (Hatosy and Martiny 2015), marine sediments (Rahman *et al*. 2008) and the polar environment (Rahman *et al*. 2015). Since ARGs are retained even under the very low selective pressures (Gullberg *et al*. 2011), it has been suggested that ARGs are disseminated widely and persist in most environments. Unlike chemical pollutants, which do not multiply in the environment, genetic pollutants such as ARGs, originating both from natural and man-made settings, can be replicated and increase in their abundance in bacterial communities in the environment.

Among various genetically polluted environments, water environments are the most probable, representing huge ARG reservoirs into which clinical and terrestrial bacteria flow and in which diverse human commensal bacteria thrive. Humans and animals are readily exposed to the ARG-possessing bacteria in the water environment, a situation that has been described metaphorically as a ‘bazaar’ (Suzuki and Hoa 2012). Bacteria colonise various substrate surfaces (Hall-Stoodley, Costerton and Stoodley 2004), forming multispecies microbial communities referred to as biofilms (Besemer 2015). In water environments, biofilms are found in many contexts such as rock surfaces, water treatment systems, hot springs, microplastics and so on (Oberbeckmann *et al*. 2014; Besemer 2015; Michels *et al*. 2018). ARGs including tetracycline resistance genes, for example *tet*(M) and *tet*(S), and sulphonamide resistance genes *sul1–sul3* are found in various water environments such as sea water and sediments, aquacultures and fish (Kim, Nonaka and Suzuki 2004; Nonaka, Ikeno and Suzuki 2007; Hoa *et al*. 2008; Suzuki *et al*. 2019); these ARGs tend to migrate downstream and accumulate in biofilms (Engemann *et al*. 2008; Zhang *et al*. 2009; Balcazar, Subirats and Borrego 2015; Guo *et al*. 2018).

Horizontal gene transfer (HGT) and gene exchange are the motive forces for dissemination of ARGs (Aminov 2011). Conjugation is presumably the principal route of HGT in bacterial communities, and the conjugation elements such as conjugative plasmids often harbour multiple ARGs (Wozniak and Waldor 2010). Goodman *et al*. (1993) and many other researchers demonstrated that conjugation occurs under simulated marine environment conditions or oligotrophic conditions. Angles, Marshall and Goodman (1993) revealed that the transfer frequency is higher between cells in biofilms attached to glass beads than between cells in the aqueous phase. Hence, biofilms are thought to be the main ARG reservoirs to proliferate ARGs and ARB in the aquatic environments. ARBs that detached themselves from biofilms can spread in the environments and may pose a threat to human health.

Membrane vesicles (MVs) have recently been reported to be abundant biological entities that are carrying environmental DNA in ocean (Biller *et al*. 2014), with the potential to transfer genes horizontally (Chiura *et al*. 2011). We hypothesise here that biofilms and MVs constitute the huge ARG reservoir in aquatic environments and that they play the important role in ARG's exchange. In this review, we focus on HGT mechanisms including the MV-mediated gene transfer and the interconnections of HGT and biofilms. We will describe first the general explanation for biofilm development, and then recent progress in studying HGT mechanisms while paying attention to the role of MVs. We will also review the methodological developments, and discuss the future challenges to fill gaps in our understanding of HGT in biofilms.

## BIOFILM FORMATION

Bacteria classically have been considered unicellular organisms, but in nature, they prefer to form highly structured multicellular communities, termed biofilms, to survive in harsh environments (Flemming *et al*. 2016; Toyofuku *et al*. 2016). Diverse bacterial species can live together in close proximity in biofilms, where the cells show remarkable and distinct features that are not seen in their planktonic form: heterogeneity of gene expression, division of roles in the community and enhanced tolerance to antibiotics (Hall and Mah 2017). Bacterial cells in biofilms are embedded at a high density within a matrix of extracellular polymeric substances (EPSs). EPSs are biopolymers produced by the cells within biofilms, and typically are composed of exopolysaccharides, amyloid-like proteins, lipids and extracellular DNA (eDNA) (Fulaz *et al*. 2019). Individual steps in biofilm formation have been classified into attachment, microcolony formation, maturation and detachment stages (Toyofuku *et al*. 2016; Guilhen, Forestier and Balestrino 2017). Time scale for the biofilm development differs between bacterial species, and heavily depends on the culture conditions. According to *in situ* observation of marine biofilms at a coastal area in southern Baltic Sea, microcolonies on the solid surface reached to the mature stage in 20–25 days (Grzegorczyk *et al*. 2018). At the beginning of the biofilm formation, planktonic cells attach to solid surfaces (for development of adhesive biofilms) or gather at an air–liquid interface (floating or pellicular biofilms). Then, the cells propagate and aggregate to form microcolonies consisting of small number of the cells. During microcolony formation, the cells begin to produce EPSs, which support the attachment and aggregation of cells, and serve as a scaffold for biofilms. EPSs are the major component of biofilms, accounting for over 90% of the biofilm mass (Fulaz *et al*. 2019). EPS production is controlled by complex genetic regulation and environmental factors such as nutrients and temperature (Cairns, Hobley and Stanley-Wall 2014; Obana, Nakamura and Nomura 2014; Toyofuku *et al*. 2016). Cellular components from dead cells also are utilised to stabilise the biofilm structure. In particular, eDNA released from lysed cells within the biofilm is an important source of EPSs (Das, Sehar and Manefield 2013; Ibanez de Aldecoa, Zafra and Gonzalez-Pastor 2017; Fulaz *et al*. 2019). Microcolonies develop into mature biofilms through further cell growth and EPS production. During the development and maintenance of biofilms, some cells detach themselves from the biofilms due to external and internal factors (Toyofuku *et al*. 2016; Guilhen, Forestier and Balestrino 2017); for instance, parts of biofilms can be torn apart by external physical forces such as friction, pressure and rapid water flow. On the other hand, subpopulations within biofilms can turn into motile cells or into producers of EPS-degrading enzymes by switching gene expression in response to inter- and intracellular signals and environmental changes. The detached cells then revert to their planktonic form, travelling to find new niches.

## MECHANISMS OF HGT IN BIOFILMS

**Figure 1.**
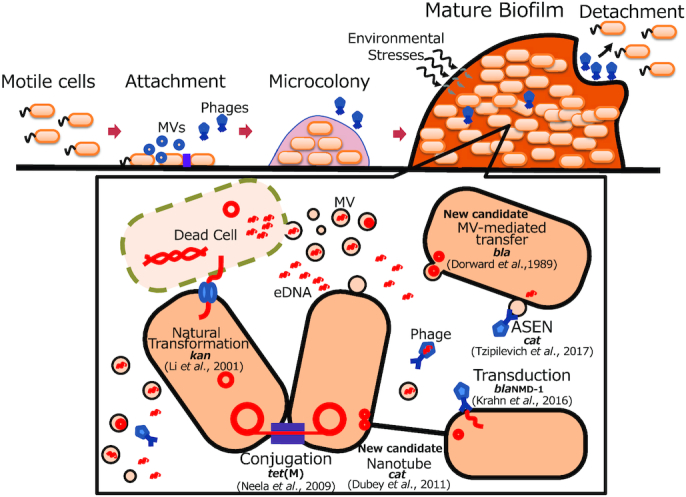
Biofilm life cycle and HGT. The typical life cycle of biofilms includes attachment to surfaces, microcolony formation, maturation and detachment. MGE-encoded factors and MVs enhance attachment and cell aggregation at the early stages of biofilm formation. Degradation of the biofilm matrix by phages facilitates cell detachment. ARGs can be distributed via three classical mechanisms: conjugation, natural transformation and phage transduction (and ASEN), and two candidates for new HGT mechanisms: MVs and nanotube (denoted as new candidate) in biofilms. Examples of ARGs transferred through each pathway are shown with the respective references. DNA is depicted by red lines and circles.

Many studies have shown that bacteria frequently exchange ARGs in biofilms (Table 1; Molin and Tolker-Nielsen 2003; Balcazar, Subirats and Borrego 2015). ARGs often are encoded in mobile genetic elements (MGEs) such as conjugative and non-conjugative plasmids (Carattoli 2013), transposons (Partridge *et al*. 2018), integrative and conjugative elements (ICEs) (Wozniak and Waldor 2010) and bacteriophages (Calero-Caceres, Ye and Balcazar 2019). ARGs reside in accessory regions of the MGEs, that is, regions that are not necessary for the maintenance and mobilisation of the elements (Partridge *et al*. 2018). HGT is thought to be driven by three major mechanisms: conjugation, natural transformation and bacteriophage infection. The types of DNA transferred largely depend on the HGT mechanisms: conjugative plasmids and ICEs are transferred via conjugation, chromosomal DNA and non-conjugative plasmids via transformation, and bacteriophage genomic DNA via infection. In addition to these classic mechanisms of HGT, MVs, which are abundant DNA reservoirs in aquatic environments (Biller *et al*. 2014), have the potential to transfer genes between bacteria. Figure 1 provides a schematic summary of the major well-studied routes of HGT and newly notable mechanisms along with the typical life cycle of biofilms. Examples of studies on HGT of ARGs are listed in Table 1.

**Table 1.** Examples of studies on HGT of ARGs

Donors/recipients	HGT mechanisms	Genetic materials	Transferred ARGs	Conditions/method	Remarks	References
*P. damselae/E. coli*	Conjugation	pAQU1	*tet*(M), *tet*(B), *sul2, floR, bla_CARB-9_*-like, *mph*(G), *mef*(C)	Filter mating		Nonaka *et al*. (2012)
*S. fidelis, P. damselae/E. coli*	Conjugation	pAQU1-like	*tet*(M), *tet*(B), *sul2, floR, bla_CARB-9_*-like, *mph*(G), *mef*(C)	Filter mating		Nonaka *et al*. (2014)
*S. aureus/S. aureus*	Conjugation	pGO1	*dfrA, aacA-aphD*	Filter mating		Savage, Chopra and O'Neill (2013)
*L. garvieae*/*E. faecalis, Vibrio*spp.*/E. coli*	Conjugation	Chromosome	*tetM*	Filter mating		Neela *et al*. (2009)
*E. coli*/environmental bacteria (e.g. *Actinobacteria, Gammaproteobacteria, Betaproteobacteria*)	Conjugation	pKJK5	*dfrA1, kan*	Biofilms, planktonic cells, filter mating	Biofilms were formed on microplastics in lake. Planktonic cells were collected from lake water.	Arias-Andres *et al*. (2018)
*V. ponticus, E. coli/E. coli*	Conjugation	pSEA1	*tet*(B), *tet*(M), *mef*(C), *mph*(G), *sul2, catII, bla*	Filter mating		Nonaka *et al*. (2018)
*V. dispar*/four streptococcal species	Conjugation, transformation	Chromosome (Tn*916*)	*tet*(M)	Biofilm in artificial saliva	Biofilms were formed on enamel matrix in artificial saliva.	Hannan *et al*. (2010)
Purified DNA*/S. mutans*	Transformation	pDL289, chromosome	*kan, erm*	Biofilm in liquid medium		Li *et al*. (2001)
Purified DNA/*Acinetobacter* sp.	Transformation	pGAR1	*tet*	Biofilm in a flow cell		Hendrickx, Hausner and Wuertz (2003)
*E. coli*(φ731)/*E. coli*	Transduction	Phage 731 DNA (*stx*::*cat*)	*cat*	Biofilm in liquid medium		Solheim *et al*. (2013)
*C. difficile*(φC2)/*C.difficile*	Transduction	Chromosome (Tn*6215*)	*erm(B)*	Planktonic cells in liquid medium		Goh *et al*. (2013)
*A. baumannii* (φR2090-I, II, III)/*A. baumannii*	Transduction	Chromosome (Tn*125*)	*bla_NDM-1_*	Planktonic cells in liquid medium		Krahn *et al*. (2016)
*B. subtilis/B. subtilis*	Nanotube	pHB201	*cat, erm*	Colony on agar plate	This is the single evidence of nanotube-mediated HGT of ARGs.	Dubey and Ben-Yehuda (2011)
*N. gonorrhoeae* MV/*N. gonorrhoeae*	MV	R1	*bla*	Planktonic cells in liquid medium	This is the first report of MV-mediated gene transfer.	Dorward, Garon and Judd (1989)
*E. coli* MV/*E. coli, S.enterica*	MV	pGFP	*bla*	Planktonic cells in liquid medium		Yaron *et al*. (2000)
*A. baylyi* MV/*A. baylyi, E. coli*	MV	pMU125	*bla*	Planktonic cells in liquid medium		Fulsundar *et al*. (2014)
*A. baumannii* MV*/A. baumannii*	MV	pMMA2, pMMCU3	*bla_OXA-2_*	Planktonic cells in liquid medium		Rumbo *et al*. (2011)
*B. agrestis* MV*/B. agrestis*	MV	pBBRMCS-1	*cat*	Planktonic cells in liquid medium	The authors mentioned specie-specific interaction between MVs and bacterial cells.	Tashiro *et al*. (2017)
*Thermus* MV/*Thermus* spp.	MV	pMKPnqosyfp	*kan*	Planktonic cells in liquid medium		Blesa and Berenguer (2015)
*A. eronii, E. cloacae, E. coli* MVs/*A. veronii, E. cloacae, E. coli, P. aeruginosa*	MV	pLC291	*kan*	Planktonic cells in liquid medium	MV-mediated HGT between four bacterial species	Tran and Boedicker (2017)
*Thermus* spp. MVs/*Thermus* spp.	MV	Chromosome	*kan*	Planktonic cells in liquid medium		Blesa and Berenguer (2015)
*P. gingivalis* MV/*P. gingivalis*	MV	Chromosome	*ermF, ermAM*	Planktonic cells in liquid medium		Ho *et al*. (2015)
SPP1 phage*/* SPP1-resistant *B. subtilis*	ASEN (acquisition of sensitivity; MV & phage)	SPP1 phage (pBT163)	*cat*	Planktonic cells in liquid medium	This is the first report of ASEN.	Tzipilevich, Habusha and Ben-Yehuda (2017)

### Conjugation

Conjugation transfers ICEs and conjugative plasmids through a proteinaceous apparatus, the conjugation pilus, that serves as a physical link between the donor and recipient cells (Partridge *et al*. 2018). Conjugation is a powerful mechanism to spread ARGs in biofilms due to conjugation delivery of DNA beyond bacterial species. In addition, conjugation elements often carry multiple ARGs: for example, a 204-kb conjugative plasmid pAQU1 isolated from a coastal aquaculture in Japan (Nonaka *et al*. 2012) carries *tet*(M), *tet*(B), *sul2, floR*, a β-lactamase (*bla_CARB-9_*-like) gene, and macrolide resistance genes *mph*(G) and *mef*(C). Its relative plasmids and ARGs were also found in various aquatic environments in Japan, Taiwan and Thailand (Nonaka *et al*. 2014; Sugimoto *et al*. 2017). As another example, pB10, a 64-kb broad-host-range conjugative plasmid, which was isolated from a wastewater treatment plant, harbours *tetA, sul1*, an amoxicillin resistance gene (*oxa2*), streptomycin resistance genes (*strA* and *strB*) and mercury resistance genes (*merA, merD, merE, merP* and *merT*) (Schluter *et al*. 2003).

Conjugation rarely occurs between motile planktonic cells because the transfer requires a direct contact between the donor and recipient cells. However, this disadvantage is overcome in biofilms, where cells are attached to a matrix and located close together for a more-extended interval. Indeed, many reports indicate that biofilms enhance conjugation (Molin and Tolker-Nielsen 2003; Madsen *et al*. 2012). Under laboratory conditions, *Staphylococcus aureus* biofilms increased the transfer rate of a conjugative plasmid (pGO1) carrying trimethoprim and gentamicin resistance genes, providing increases in transfer rates of up to ∼16 000-fold compared to planktonic cells (Savage, Chopra and O'Neill 2013).

Under aquatic conditions, the horizontal transfer of a conjugative plasmid (pKJK5) harbouring a trimethoprim resistance gene (*dfrA1*) and a *tetA* was shown within microplastic-localised biofilms composed of diverse species in lake water (Arias-Andres *et al*. 2018). Neela *et al*. (2009) reported that *tet*(M) was transferred from marine *Lactococcus garvieae* to human *Enterococcus faecalis*, but not to *Escherichia coli*. In contrast, *Vibrio* spp. transferred *tet*(M) to *E. coli*, but not to *E. faecalis*. These donors (*L. garvieae* and *Vibrio* spp.) are fish-pathogenic bacteria; *in vivo*, these organisms would form biofilms on fish intestine, where the transfer of ARGs would occur. Additionally, biofilms enhanced the persistence of pKJK5 (Bahl, Hansen and Sorensen 2007) and ARGs (Zhang *et al*. 2009) in the absence of selective pressure. Persistence of tetracycline resistance genes has also been reported in sea farms even in the absence of selective pressure (Tamminen *et al*. 2011). Laboratory experiments showed that pAQU1 and the related plasmids are stable within the community of non-culturable bacteria in sterile seawater and well water without selective pressure, where the cells are in a state of deep dormancy in response to adverse environments such as severe starvation (Bien *et al*. 2015). Once a multidrug resistance plasmid is introduced into the community, the episome should persist stably in the environmental bacterial community even during grazing by protists (Bien *et al*. 2017).

### Nanotube

Recently, an elongated extracellular structure, named the nanotube, was discovered as another mechanism of DNA transfer; nanotubes are employed in direct cell-to-cell contact in *Bacillus subtilis* (Dubey and Ben-Yehuda 2011; Dubey *et al*. 2016). Nanotubes are membranous structures, and they are distinguishable from conjugation pili, which are composed of proteins. Nanotubes were shown to transport a non-conjugative plasmid (pHB201) carrying a chloramphenicol resistance gene (*cat*) and an erythromycin resistance gene (*erm*) between *B. subtilis* cells (Dubey and Ben-Yehuda 2011), although there is no evidence of HGT mediation through nanotubes in aquatic environments. Nanotube-like structures have been described for various species including *E. coli, Acinetobacter baylyi, Desulfovibrio vulgaris* and *Clostridium acetobutylicum* (Benomar *et al*. 2015; Pande *et al*. 2015; Baidya *et al*. 2018). Unlike conjugation pili that transfer DNA associated with the relaxosome proteins, nanotube (-like) structures are capable of transporting cytoplasmic components such as nutrients and fluorescent marker proteins, as well as DNA. Therefore, nanotubes likely play a significant role in distribution of biomaterials (beyond ARGs alone) within bacterial communities.

### Natural transformation

Natural transformation is a genetic alteration mediated by uptake of exogenous DNA through the competence machinery consisting of a transformation pilus and a DNA transporter (Lorenz and Wackernagel 1994). DNA incorporated through the machinery is integrated into the bacterial chromosome by homologous recombination, or the introduced DNA is autonomously replicated if able to function as an episome. DNA transfer via this mechanism absolutely relies on bacterial species with the ability to develop DNA competence. A total of 82 species, including *Streptococcus pneumoniae, B. subtilis* and *Vibrio cholerae*, are now known to be naturally transformable (Johnston *et al*. 2014). Unlike conjugation, transformation does not require a physical contact between the donor and recipient cells. Free DNA released by cell lysis can serve as the donor for transformation. Hannan *et al*. (2010) showed that a conjugative transposon (Tn*916*) carrying *tet*(M) was transferred from *Vellonella dispar* living cells to four different streptococcal species via conjugation in their biofilms; even purified naked *V. dispar* DNA containing Tn*916* was able to serve as a donor for transformation.

In aquatic biofilms, *Streptococcus mutans* cells were naturally transformed by addition of a plasmid (pDL289) encoding a kanamycin resistance gene (Li *et al*. 2001). *Acinetobacter* sp. BD413 biofilms formed in LB medium have been shown to be transformable with an exogenous plasmid (pGAR1) that carries a tetracycline resistance gene, using flow cell system (Hendrickx, Hausner and Wuertz 2003). Williams *et al*. (1996) demonstrated that *Acinetobacter* BD413 cells in river biofilms were transformable with pQM17, a mercury resistance plasmid.

### Bacteriophages

Bacteriophages (phages) are viruses that infect bacteria (Penades *et al*. 2015). Phages are important DNA reservoirs in natural environments; indeed, phages are the most abundant biological entities on the planet, and DNA packaged in phage particles are stable, avoiding digestion by nucleases. Importantly, ARGs in phages cannot be eliminated completely by wastewater disinfection treatments such as UV irradiation and chlorination (Calero-Caceres and Muniesa 2016), because phages are non-living entities and highly resistant to such treatments. Phages inject their DNA into the host cells during infection, which is a transduction gene transfer (Penades *et al*. 2015). Along with their own genomes, phages can deliver non-viral DNA derived from bacterial chromosomes, transposons and plasmids.

Some temperate phages, a subgroup of phages that insert their genomes into the host chromosome upon infection (a process called lysogenisation), possess antibiotic resistance genes in their genomes; for instance, β-lactam resistance gene *aci1* is carried by an *Acidaminococcus* phage (Rands *et al*. 2018), and the metallo β-lactamase gene *mbl* is carried by a *Veillonella* phage (Rands, Brussow and Zdobnov 2019). Lysogenisation of such phages confers antibiotic resistance upon the bacterial host. Typically, temperate phages that reside in the bacterial genome become active in response to DNA damage, at which point the lysogenised phages begin to produce progeny. By contrast, *B. subtilis* phage SPβ, carrying the probable aminoglycoside resistance gene *yokD* (Klimecka *et al*. 2011), resides within a sporulation gene (*spsM*); the phage genome is excised from the *B. subtilis* chromosome during sporulation, thereby reconstituting a functional *spsM* gene (Abe *et al*. 2014; Abe, Takamatsu and Sato 2017a). A similar behaviour is observed in many temperate phage (-like) elements in Gram-positive spore-forming bacteria (Sato, Samori and Kobayashi 1990; Abe *et al*. 2013; Abe *et al*. 2017b). The excised phage (-like) elements form a circular DNA without packaging into phage particles. Although the fate of the circular DNA remains unknown, it might be horizontally transferred via natural transformation or other pathways rather than by phage infection. Such HGT by novel mechanisms may occur in aquatic bacterial communities.

Metagenomic analyses have detected various ARGs in phage fractions isolated from environmental water samples including genes encoding resistance to aminoglycoside, β-lactam, macrolide, quinolone and sulphonamide, and tetracycline antibiotics from sewages, river water, seawater and WWTPs (Colomer-Lluch *et al*. 2014; Lekunberri *et al*. 2017a,b; Wang *et al*. 2018; Yang *et al*. 2018). Phage-mediated transfer of ARGs has been reported in many bacteria under laboratory settings (von Wintersdorff *et al*. 2016). As examples, Solheim *et al*. (2013) showed that the phage-mediated transfer of *cat* occurred inside of *E. coli* biofilms in liquid medium. A *Clostridium difficile* phage (phiC2) has been shown to deliver an erythromycin resistance gene *erm*(B) (carried by a Tn*6215* transposon) between *C. difficile* cells (Goh *et al*. 2013). Likewise, *Acinetobacter baumannii*, which is an important multidrug-resistant human pathogen, has been shown to transfer *bla_NDM-1_* (carried within a Tn*125* transposon) via phage transduction (Krahn *et al*. 2016). This bacterium is a commensal species in water, suggesting that *A. baumannii* is capable of transporting ARGs between the natural aqueous and man-made environments.

### MV-mediated exchange of ARGs

MVs are typically 20- to 400-nm-diameter lipid-bilayer-enclosed particles released from bacteria (Toyofuku *et al*. 2015, 2019). MVs originally were reported in the 1960s, when their release was observed following outer membrane blebbing in Gram-negative bacteria (Brown *et al*. 2015; Toyofuku *et al*. 2015); however, recent work has shown that Gram-positive bacteria also produce MVs (Brown *et al*. 2015; Sugimoto *et al*. 2016; Toyofuku *et al*. 2017b; Toyofuku, Nomura and Eberl 2019). MVs are released not only from planktonic cells but also within biofilms. MV production in biofilms has been reported in *Pseudomonas aeruginosa* (Murphy *et al*. 2014), *Helicobacter pylori* (Yonezawa *et al*. 2009), *E. coli* (Nakao *et al*. 2018), *B. subtilis* (Brown *et al*. 2014) and *S. aureus* (Sugimoto *et al*. 2016).

The first report of the MV-mediated gene delivery was transfer of a R-plasmid carrying *bla* in *Neisseria gonorrhoeae* (Dorward, Garon and Judd 1989). Since that first finding, many laboratory studies have demonstrated the MV-mediated mobilisation of ARGs in a wide range of bacteria (Domingues and Nielsen 2017), although MV-mediated HGT in natural environments remains to be proven. MV-mediated transfer of ARGs carried by plasmids was reported in experiments with *E. coli* and *Salmonella enterica* using *bla* in pGFP (Yaron *et al*. 2000), in *A. baylyi* with *bla* in pMU125 (Fulsundar *et al*. 2014), in *A. baumannii* with *bla*_OXA-2_ in pMMA2 and pMMCU3 (Rumbo *et al*. 2011), in *Buttiauxella agrestis* with a chloramphenicol resistance gene in pBBRMCS-1 (Tashiro *et al*. 2017) and in *Thermus* spp. with *kan* in pMKPnqos*yfp* (Blesa and Berenguer 2015). Moreover, interspecies plasmid transfer via MVs was reported from *Aeromonas veronii, Enterobacter cloacae* and *E. coli* donors into *A. veronii, E. cloacae, E. coli, P. aeruginosa* recipients; transfer was detected using pLC291, a broad-range plasmid that carries *kan* (Tran and Boedicker 2017). MV-mediated transfer of chromosomal DNA containing ARGs has been shown for *kan* in *Thermus* spp. (Blesa and Berenguer 2015) and for *ermF* and *ermAM* in *Porphyromonas gingivalis* (Ho *et al*. 2015). Interestingly, MVs are capable of conveying quorum-sensing (QS) signals in Gram-negative bacteria such as *P. aeruginosa* (Mashburn and Whiteley 2005), *Paracoccus* sp. (Toyofuku *et al*. 2017a; Morinaga *et al*. 2018) and *Vibrio* sp. (Brameyer *et al*. 2018). QS signals are known to regulate conjugation (Piper, Beck von Bodman and Farrand 1993), transformation (Suckow, Seitz and Blokesch 2011) and phage induction (Laganenka *et al*. 2019). Therefore, MVs may be involved in regulation of HGT, as well as DNA transportation.

MVs are ubiquitous and are abundant in seawater samples (∼6 × 10^6^ and ∼3 × 10^5^ particles/mL in coastal surface water and Sargasso seawater samples, respectively; Biller *et al*. 2014). MVs isolated from the seawater samples contained a diverse pool of DNA with significant homology to members of 33 phyla including *Proteobacteria, Cyanobacteria, Bacteroidetes* and *Firmicutes* (Biller *et al*. 2014). MVs are also found in river water (Roose-Amsaleg *et al*. 2017). Chiura *et al*. (2011) demonstrated that MVs collected from seawater were capable to transfer auxotrophic marker DNA to *E. coli* in the laboratory experiment. However, there is no study that proves the MV-mediated HGT in biofilms. Further study will be required to show the direct evidence.

## INTERCONNECTIONS OF HGT MECHANISMS AND BIOFILMS

Some conjugative plasmids facilitate biofilm development by encoding biofilm-associated proteins. pCF10, an *E. faecalis* conjugative plasmid, encodes three cell-wall-anchoring proteins (PrgA, PrgB and PrgC) that promote cell–cell adhesion at an early stage of biofilm formation (Bhatty *et al*. 2015). pOLA52, a *Klebsiella pneumonia* plasmid, possesses genes encoding type III fimbriae, which are involved in cell attachment to surfaces (Burmolle *et al*. 2008). *Escherichia coli* has many conjugative plasmids, including, for example, the F plasmid, which promotes biofilm formation in a conjugation-pilus-dependent manner (Ghigo 2001).

Natural transformation is known to be closely connected with biofilm formation in streptococci and *V. cholerae* (Ibanez de Aldecoa, Zafra and Gonzalez-Pastor 2017; Veening and Blokesch 2017); eDNA, which is a major component of the biofilm matrix, is released during the development of DNA competence. Streptococcal species (e.g. *S. pneumoniae* and *S. mutans*) exhibit a phenomenon called fratricide. The competent cells increase production of extracellular cell-wall degrading enzymes and bacteriocins, causing lysis of neighbouring cells and release of eDNA (Steinmoen, Knutsen and Havarstein 2002; Moscoso and Claverys 2004). Oggioni *et al*. (2006) showed that the addition of artificially synthesised competence-stimulating peptide (CSP) promotes *S. pneumoniae* biofilm formation, whereas no biofilm was formed by CSP receptor mutants. In *S. mutans*, the transformation efficiency correlates with development of the biofilm (Li *et al*. 2001). As is the case in streptococci, *V. cholerae* competent cells kill neighbouring cells by injection of effector proteins through a type VI pilus, leading to recipient cell death and release of eDNA (Veening and Blokesch 2017). In addition to the role in eDNA production, the *V. cholerae* competence pilus itself promotes cell aggregations via pilus–pilus interaction at the early stage of the biofilm formation (Adams *et al*. 2019).

Phages invade biofilms by disrupting the matrix and killing the embedded cells (Sutherland *et al*. 2004). Apparently, biofilm formation and phages are mutually exclusive. However, recent work has illustrated the positive role that phages can play in supporting the life cycle of biofilms. In many cases, phage-mediated cell lysis leads to production of eDNA, which strengthens the biofilm structures (Fernandez, Rodriguez and Garcia 2018). In another case, destruction of biofilms by the *E. coli* phage Rac (Liu *et al*. 2015) and the *P. aeruginosa* phage Pf4 (Rice *et al*. 2009) results in detachment of the cells from biofilms. Moreover, the *P. aeruginosa* prophage Pf4 also is involved in the stabilisation of microcolonies, thereby shaping the mature biofilm structure, and in virulence in mice (Rice *et al*. 2009).

MVs contribute to the development of biofilms in *H. pylori* (Yonezawa *et al*. 2009), *V. cholerae* (Altindis, Fu and Mekalanos 2014) and *Pseudomonas putida* (Baumgarten *et al*. 2012); MVs promote adhesion of cells to surfaces and/or cell aggregation at the early stages of biofilm formation, probably by increasing the cell surface hydrophobicity. Perhaps the most significant feature of MVs is that these structures can contain and transport various biomolecules such as DNA, RNA, proteins, metabolites and QS signals, thereby participating in many physiological processes including gene transfer, virulence, nutrient acquisition, cell defence and cell–cell communication (Tashiro, Uchiyama and Nomura 2012; Toyofuku *et al*. 2015). Importantly, cargos in MVs such as DNA and proteins exhibit resistance to extracellular enzymes (nucleases and proteinases) that would normally degrade these substrates (Toyofuku *et al*. 2015). Because of their ability to carry DNA and their abundance in nature, MVs now are gathering attention as potential agents of HGT (Domingues and Nielsen 2017).

MVs are known to modulate interactions between bacteria and phages. *Bacillus subtilis* SPP1 phage-resistant cells, which lack the SPP1 receptor protein, became susceptible to the phage when they captured MVs containing the receptor, leading to transduction of pBT163, a *cat*-encoding plasmid (Tzipilevich, Habusha and Ben-Yehuda 2017). This phenomenon, named ASEN (acquisition of sensitivity), may cause expansion of phage infection in bacterial communities; however, at the same time, it is likely to contribute to phage-mediated HGT beyond the host-range limitation. The relationship between MVs and phage is not limited to the modulation of phage susceptibility. Lysogenised phages play a critical role in the MV production in bacterial communities. Cell lysis caused by phage-derived lytic enzymes releases MVs in *P. aeruginosa* (Toyofuku *et al*. 2014; Turnbull *et al*. 2016) and *B. subtilis* (Toyofuku *et al*. 2017b).

## METHODOLOGIES FOR STUDYING HGT AND BIOFILMS

From the past to the present, detection of ARGs and ARB has been generally performed by quantitative PCR using environmental DNA and genomic analysis of cultivated clonal ARB isolated from the environments. Filter mating, transformation and transduction assays are often used to verify the transfer of ARGs in laboratories (Table 1). Currently, high-throughput next-generation DNA sequencers (NGS) provide vast amounts of whole-genome data of organisms, and NGS allow researchers to obtain multispecies genomic data directly from uncultivated bacteria in the natural environments (metagenome) (Bragg and Tyson 2014). The sequenced data are deposited on the public databases [e.g. comprehensive ARG database, CARD, (McArthur *et al*. 2013)] and available for further analysis, such as classifying ARGs and identifying HGT events. To date, many computational pipelines and software have been developed to detect HGT (Douglas and Langille 2019). In particular, Song *et al*. (2019) and Li, Jiang and Li (2019) have recently created MetaCHIP and LEMON, respectively, which are aimed for prediction of HGT events in bacterial communities from metagenomic data. These authors mentioned the availability of these software to detect mobilisation of ARGs. Utilisation of the software may provide information of how ARGs have been transferred in the past within the individual microbial communities. Although the recent environmental ARG research may largely rely on the cultivation-independent metagenomic analysis, the cultivation-based approach is still needed to study antibiotic resistance properties of newly identified ARBs and mobilisation of novel MGEs, whose information is not obtained only from the sequencing data.

Combinations of experimental and bioinformatic methodologies contribute to discovery of new HGT pathways and mechanisms beyond detection of ARGs. For examples, Jiang *et al*. (2017) and Nonaka *et al*. (2018) employed integrative approaches to examine interspecies transfer of ARGs through combination reactions of transposase/integrase-mediated transposition and homologous recombination. Jiang*et al*. (2017) identified potential examples of ARGs within transposons transferred from actinobacteria to proteobacteria by informatic analysis of genomic sequences deposited in public databases, and then experimentally confirmed the mobilisation. Their results suggest an explanation for the emergence of antibiotic-resistant pathogens through interspecies HGT. Nonaka *et al*. (2018) examined the conjugative transfer of a multidrug resistance plasmid (pSEA1) between *E. coli* and *Vibrio ponticus*. Whole-genome sequencing of the transconjugant and subsequent molecular genetic analysis revealed the two-step mechanism underlying the interspecies transfer of pSEA1, where during conjugation of thepSEA1, Tn*6283* on the plasmid first transposes into the recipient genome and consequently, another transferred pSEA1 can be integrated into the genome through a homologue recombination event at the Tn*6283* sequences between the recipient genome and pSEA1. These examples demonstrate the usefulness of the combination approaches.

For simulations of ARGs dissemination, mathematical models for HGT have been devised (Nielsen and Townsend 2004; Sørensen *et al*. 2005; Mao and Lu 2016). Recently, Nazarian, Tran and Boedicker (2018) reported a new computational model for HGT in multispecies bacterial communities, taking MV-mediated gene transfer into account, as well as the traditional mechanisms, conjugation, transformation and transduction. Computational simulations should be important to forecast the ARGs dissemination in the environments.

Visualisation of biofilms with microscopies is essential to understand their structures and properties. Schwartz *et al*. (2009) reported the imaging and characterisation of natural biofilms on water filter materials by use of scanning and transmission electron microscopy (SEM and TEM) and Raman microspectroscopy. Sugimoto *et al*. (2016) developed atmospheric scanning electron microscopy (ASEM) to observe nanostructures within biofilms in liquid. Confocal microscopy is useful to observe dynamics of viable and developing biofilms. Microfluidics devices combined to confocal microscopy constitutes a powerful tool to observe viable biofilms under non-invasive conditions (Christensen *et al*. 1998; Yawata, Nomura and Uchiyama 2008; Kiyokawa *et al*. 2017). Not only laboratory observation, Grzegorczyk *et al*. (2018) also developed the *in situ* observation strategy for biofilms growing in marine environments.

Li *et al*. (2018) and Qiu *et al*. (2018) reported the novel integrative microfluidic systems, which consist of microfluidics, laser confocal microscopy and flow cytometry. By use of *gfp*-carrying conjugative plasmids as donor DNA, their systems are capable of *in situ* tracking HGT of the plasmid within viable biofilms on the microfluidics devices, which allow temporal and spatial analysis of HGT in 3D biofilms and quantification of the HGT rate by counting transconjugants by following flow cytometric analysis. They succeeded in calculation of real-time HGT rates within activated sludge biofilms, suggesting that their systems are applicable for determination of HGT rates within biofilms collected from various environments, using conjugative and non-conjugative plasmids, phages and MVs as donors. Furthermore, if combined with a cell sorter and NGS, the HGT pathway may also be analysable in the system.

Since the discovery of MVs, TEM and SEM have been routinely used for analysis on their structures. In addition, the use of recent high-resolution confocal microscopy has enabled *in vivo* observation of MV release from bacterial cells (Turnbull *et al*. 2016; Toyofuku *et al*. 2017b); however, for observation of their movements in solution, high-speed and -resolution microscope technology will be needed. Moreover, little is known about the behaviour of MVs in biofilms. Future research will require more detailed analysis of the biogenesis/absorption of MVs by bacteria and the development of imaging technology allowing the *in vivo* tracking of MVs in liquid and in biofilms.

## FUTURE CHALLENGES

Evidence that the aquatic environment is a huge reservoir of ARGs is increasing (Zhang, Zhang and Fang 2009; Amos *et al*. 2014). For the risk assessment of the ARB emergence in the aquatic environments, investigation of the ARG transfer mechanisms, rates and pathways is required urgently; however, due to the complexity of the multiple HGT mechanisms and experimental limitations, it remains a challenging problem. We emphasise that closer integration of experimental and computational approaches will be needed more to establish the comprehensive strategy. Metagenomic analysis of water environments provides information on the current status of the ARGs and ARB dissemination in the environment. Analysis of the metagenomic data with the HGT detection software (e.g. MetaCHIP and LEMON) will be helpful to understand the history of ARG transfer. The latest microfluidic systems combined with laser confocal microscopy, fluorescent labelling of bacteria and flow cytometry (Li *et al*. 2018; Qiu *et al*. 2018) can provide us the *in situ* information of the quantitative rates of the ongoing HGT events in viable biofilms collected from environments. Further, if followed by cell sorting and metagenomic sequencing, it may be also possible to reveal the ARG transfer pathway in the biofilms. Such systematic experimental methodology will provide accurate and quantitative data of HGT enough to build reliable mathematic models and computational simulations, which enables us to predict the ARG dissemination in natural environments. The integrative approaches will provide a better understanding of the chronological distribution of ARGs and evolution of MGEs through complicated HGT processes.

We have highlighted in this review the previous studies that demonstrated the importance and potential of MVs as the HGT agents; however, their biological properties are still not understood fully. A very important and interesting open question is whether ARGs are exchanged through MVs between spatially separated biofilms in aquatic environments. If it is verified, ocean will be considered as the hugest genetic reservoir, where ARGs can be exchanged globally.

Water environments are very complex environments, in which biofilms are the most probable micro-hot spot of HGT of ARGs. Experimental and bioinformatic approaches are facilitating the accumulation of new evidence regarding HGT mechanisms involving ARGs, which should continue to contribute to future progress in ARB research.

## FUNDING

This work was supported in part by grants from KAKENHI (Nos. 16H01782, 16H06382 and 19K05762), JSPS, ERATO (No. JPMJER1502) and JST.
